# Augmented reality navigation for minimally invasive craniosynostosis surgery: a phantom study

**DOI:** 10.1007/s11548-022-02634-y

**Published:** 2022-05-04

**Authors:** Abdullah Thabit, Mohamed Benmahdjoub, Marie-Lise C. van Veelen, Wiro J. Niessen, Eppo B. Wolvius, Theo van Walsum

**Affiliations:** 1 Department of Radiology and Nuclear Medicine, Erasmus MC, University Medical Center Rotterdam, Rotterdam, The Netherlands; 2 Department of Oral and Maxillofacial Surgery, Erasmus MC, University Medical Center Rotterdam, Rotterdam, The Netherlands; 3 Department of Neurosurgery, Erasmus MC, University Medical Center Rotterdam, Rotterdam, The Netherlands; 4 Department of Imaging Physics, Faculty of Applied Sciences, Delft University of Technology, Delft, The Netherlands

**Keywords:** Augmented reality, Craniosynostosis, Craniectomy, Cranial sutures, Surgical navigation, Image guidance

## Abstract

**Purpose:**

In minimally invasive spring-assisted craniectomy, surgeons plan the surgery by manually locating the cranial sutures. However, this approach is prone to error. Augmented reality (AR) could be used to visualize the cranial sutures and assist in the surgery planning. The purpose of our work is to develop an AR-based system to visualize cranial sutures, and to assess the accuracy and usability of using AR-based navigation for surgical guidance in minimally invasive spring-assisted craniectomy.

**Methods:**

An AR system was developed that consists of an electromagnetic tracking system linked with a Microsoft HoloLens. The system was used to conduct a study with two skull phantoms. For each phantom, five sutures were annotated and visualized on the skull surface. Twelve participants assessed the system. For each participant, model alignment using six anatomical landmarks was performed, followed by the participant delineation of the visualized sutures. At the end, the participants filled a system usability scale (SUS) questionnaire. For evaluation, an independent optical tracking system was used and the delineated sutures were digitized and compared to the CT-annotated sutures.

**Results:**

For a total of 120 delineated sutures, the distance of the annotated sutures to the planning reference was $2.4\pm 1.2$ mm. The average delineation time per suture was $13\pm 5$ s. For the system usability questionnaire, an average SUS score of 73 was obtained.

**Conclusion:**

The developed AR-system has good accuracy (average 2.4 mm distance) and could be used in the OR. The system can assist in the pre-planning of minimally invasive craniosynostosis surgeries to locate cranial sutures accurately instead of the traditional approach of manual palpation. Although the conducted phantom study was designed to closely reflect the clinical setup in the OR, further clinical validation of the developed system is needed and will be addressed in a future work.

**Supplementary Information:**

The online version contains supplementary material available at 10.1007/s11548-022-02634-y.

## Introduction

Craniosynostosis is the premature fusion of one or more of the cranial sutures. It is characterized by a deformed skull shape, and it is associated with an increased risk of elevated intracranial pressure. The most prevalent type of craniosynostosis is sagittal synostosis which occurs in 40–60% of the single suture synostosis cases. It is important to treat craniosynostosis as it can lead to complications affecting sensory and neurological functions [1, 2]. A surgical procedure is usually performed between the ages of 6 months and 2 years to correct the deformed head shape and allow for normal brain growth. There are three main types of procedures to treat craniosynostosis. These include complete bone remodeling, strip craniectomy, and minimally invasive procedures; either spring distraction assisted craniectomy or endoscopic strip release with molding helmet [3]. Minimally invasive procedures have been reported to be associated with less blood loss and fewer complications [4], making them the preferred approaches.

**Fig. 1:**
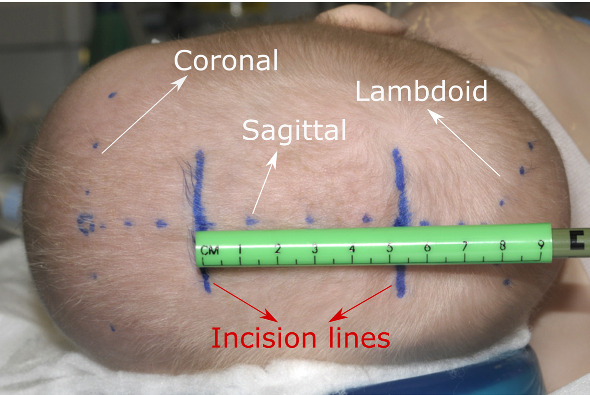
Surgery planning in minimally invasive spring-assisted craniectomy, showing the coronal, lambdoid and sagittal sutures marked by the surgeon, as well as the planned incisions. Adapted by permission from Springer Nature, Child’s Nervous System, Ref. [4], copyright 2012

In spring-assisted corrections of sagittal synostosis, the baby is first positioned on its back for surgery planning (later on its left side for the procedure) [4], where the surgeon feels the baby’s head to locate the coronal, lambdoid and sagittal sutures and mark them on the head (see Fig. 1). The surgeon then plans two incisions perpendicular to the sagittal suture at a specific distance from the coronal and lambdoid sutures. This distance is case dependent and is determined at the time of surgery (see Fig. 1). To create the planned incisions, it is therefore important to accurately locate the coronal and lambdoid sutures on the patient’s head. However, locating the sutures by feeling the head is not always accurate because of the presence of skin and hair. The error in locating the sutures using this approach could be as high as 10–20 mm, especially for the lambdoid suture that is harder to detect. This can lead to less optimal technique of the surgery and an increased surgery time.

Conventional navigation systems could potentially be used to help planning the surgery and locating the cranial sutures, but given the long setup time they need, in addition to the poor hand-eye coordination and the 2D visualization, surgeons usually choose not to use them in craniosynostosis. Augmented reality (AR) is a potential alternative for surgical navigation [5], as it can provide the suture visualization on the surgical site right in front of the surgeons’ eyes. In craniosynostosis particularly, there have been a few studies that tried to introduce AR for navigation, including Garcia-Mato et al. [6] who proposed a smartphone-based AR system that uses 3D photography and marker tracking for patient alignment. Similarly, Alshomer et al. [7] used a smartphone based AR system to assist in the planning of cranial vault reshaping. In a different study, Han et al. [8] used a camera to track a marker attached to an occlusal splint. However, smartphones or external cameras are not ergonomically convenient to use in the operating room (OR) as they require the surgeons to hold and point them at the surgical site causing a disruption in the surgery workflow. On the other hand, head-mounted displays (HMDs) offer a hands-free visualization that allow the surgeon to focus on the task at hand [9].

In this work, we propose an HMD-based AR navigation system for craniosynostosis. To that end, we develop a system that combines an electromagnetic tracking system (EMTS) with a see-through HMD. In addition, we conduct a phantom study assessing the locating accuracy of cranial sutures for surgical planning in minimally invasive spring-assisted craniectomy. We use the combined AR-EM navigation system for alignment and tracking and we ask participants to delineate the visualized sutures on two skull phantoms. For evaluation, we compare the delineation of the sutures with the planned trajectories, and we assess the usability of the system with a questionnaire.

**Fig. 2:**
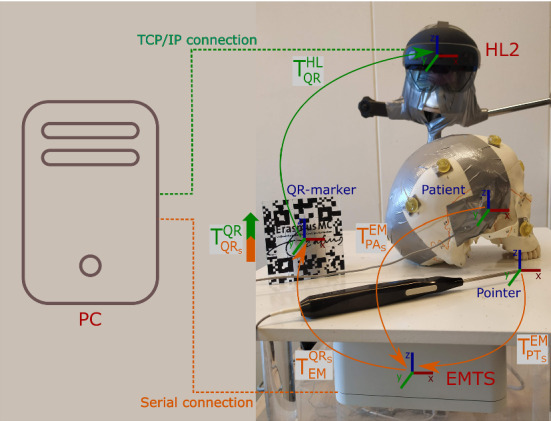
Overview of the AR-EM system: orange arrows indicate the tracking data read by the electro-magnetic tracking system (EMTS), the green arrow indicate the tracking of the QR-marker by the Microsoft HoloLens 2 (HL2), and the orange-green block arrow indicates the calibration process between the EM sensor attached to the QR-marker and the QR-marker itself. Dashed lines indicate the type of communication with the PC for both EMTS and HL2

## Methods

In this section, we describe the AR-EM navigation system developed for this study, and we outline the phantoms used, the experimental setup and the evaluation metrics utilized to assess the accuracy of the AR navigation system.

### AR navigation system

In minimally invasive corrections of sagittal synostosis, surgeons prefer not to use optical navigation systems due to their invasiveness: attaching a reference star may cause damage to the fragile skull of the child. EM navigation systems alleviate this issue by tracking electromagnetic sensors that can be taped to the child’s head. However, they still suffer from the poor hand-eye coordination and 2D visualization. Therefore, combining a reliable EMTS with an HMD for in-situ 3D visualization can provide an intuitive hand-free AR navigation.

The AR-EM navigation system consists of an EMTS (NDI Aurora, Waterloo, ON Canada) combined with a Microsoft HoloLens 2 (HL2) device (Microsoft Corporation, Redmond, USA). An overview of the systems is shown in Fig. 2. The EMTS is used to track three electromagnetic sensors: a QR-marker sensor ($QR_S$), a patient sensor ($PA_S$) and an EM-tracked pointer ($PT_S$) that is used for point-based registration. The pose of the patient sensor and the pointer are transformed from the coordinate system of the EMTS to the coordinate system of the QR-marker sensor (see Fig. 2). This sensor is rigidly attached to a QR code that can be detected by the HoloLens using Vuforia (version 9.3, http://www.developer.vuforia.com). To align the coordinate system of the EMTS with the HL2, a one-time offline calibration process was conducted to determine the rigid transformation from the coordinate system of the EM sensor attached to the QR-marker, to the coordinate system of the QR-marker itself. This calibration process is based on paired-point matching, where 24 divot-points on the QR-marker were pin-pointed using the EM pointer and matched with their counterparts on the QR-marker model. In the developed system, EM tracking data are communicated from the EMTS to a nearby PC through a serial connection, where calibration and registration transformations are calculated. Then, the poses of the patient and pointer are sent from the PC to the HoloLens through a TCP/IP connection for visualization. The source code of the system with a sample demo is publicly available and can be found at: https://gitlab.com/radiology/igit/ar/ar-em.

### Phantoms

**Fig. 3:**
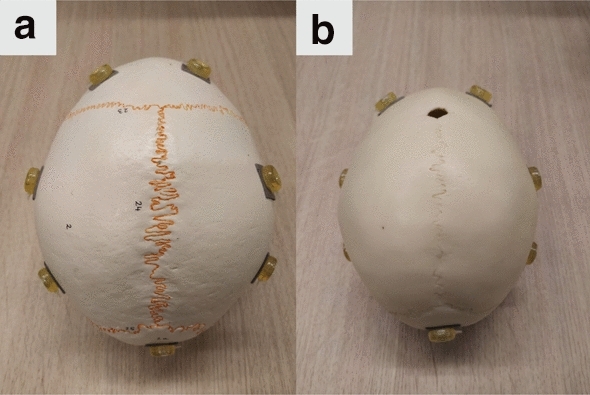
Experiment phantoms: **a** adult skull phantom SK1, **b** child skull phantom SK2

For this study, two skull phantoms were used. The first, SK1, is a skull model of an adult (Numbered Human Classic Skull Model, 3B Scientific) with good representation of the cranial sutures, see Fig. 3a. The second phantom, SK2, is a model of a child’s skull with an estimated age of around one year (14-month old Human Child Skull, Bone Clone), see Fig. 3b, where the cranial sutures are visible. The anterior fontanelle is open and the posterior is closed, which is similar to the target cases in craniosynostosis. Fiducial markers (PinPoint for Image Registration 128, Beekley) with conical shape were attached to each skull phantom: ten markers for SK1 and eight markers for SK2 as shown in Fig. 3. These markers were only used to establish a ground truth model-to-patient alignment for system evaluation.

### Preoperative planning

For preoperative planning, the two skull phantoms were CT scanned (Siemens scanner) with a voxel size of $0.2\,{\text {mm}}^3$ and resolution of $1024\times 1024\times 852$. The skull models were generated in MevisLab (MeVis Medical Solutions AG) and processed in MeshLab (www.meshlab.net) for smoothing and simplification (from two million triangles to two hundred thousands) before being finally imported to Unity (Unity Technologies) for visualization. The skull models were visualized mainly for visual check of the alignment so the viewer can ensure that the model boundaries are aligned before proceeding with sutures delineation.

**Fig. 4:**
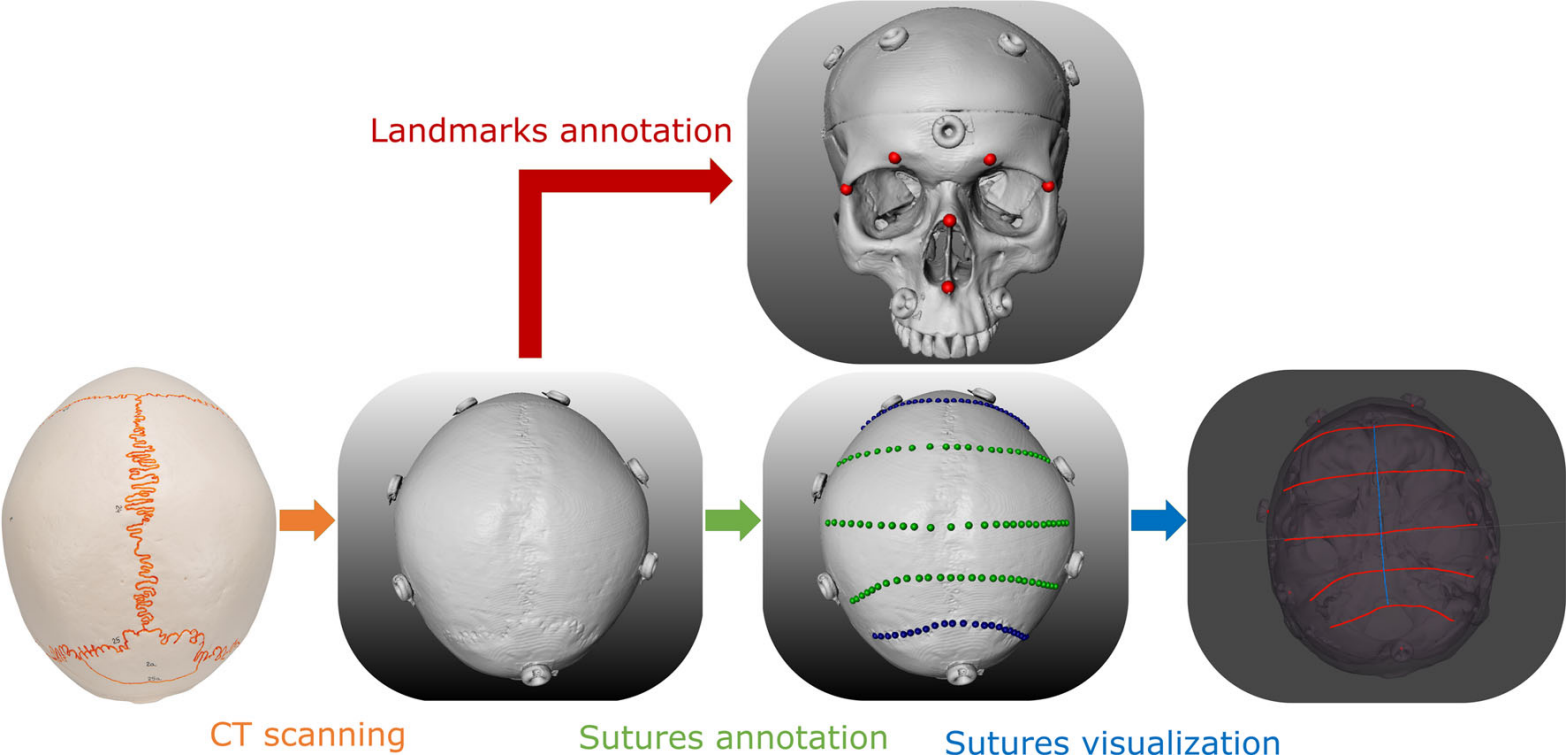
Steps in preoperative planning: phantom is CT scanned, then anatomical landmarks and suture trajectories are annotated on the CT model: landmarks (red), coronal and lambdoid sutures (blue) and virtual sutures (green), and finally the sutures are visualized in Unity

From the CT images, six anatomical landmarks (around the nose and the eyes) were identified for each skull for point-based patient-to-QR-marker alignment. Moreover, the centers of of the registration markers were identified for evaluation of the anatomical landmark registration.

For each skull, points along the coronal, lambdoid and sagittal sutures were annotated for sutures representations. In addition, three virtual sutures in between the coronal and lambdoid sutures were also annotated. These virtual sutures were created to increase the number of validating sutures and to assess the accuracy of delineation when there is no implicit prior anatomical knowledge. The suture points are then resampled at 0.25 mm and used to create the suture lines, which are imported to unity for visualization. Figure 4 shows the steps for trajectories preplanning.

### Experimental protocol

**Fig. 5:**
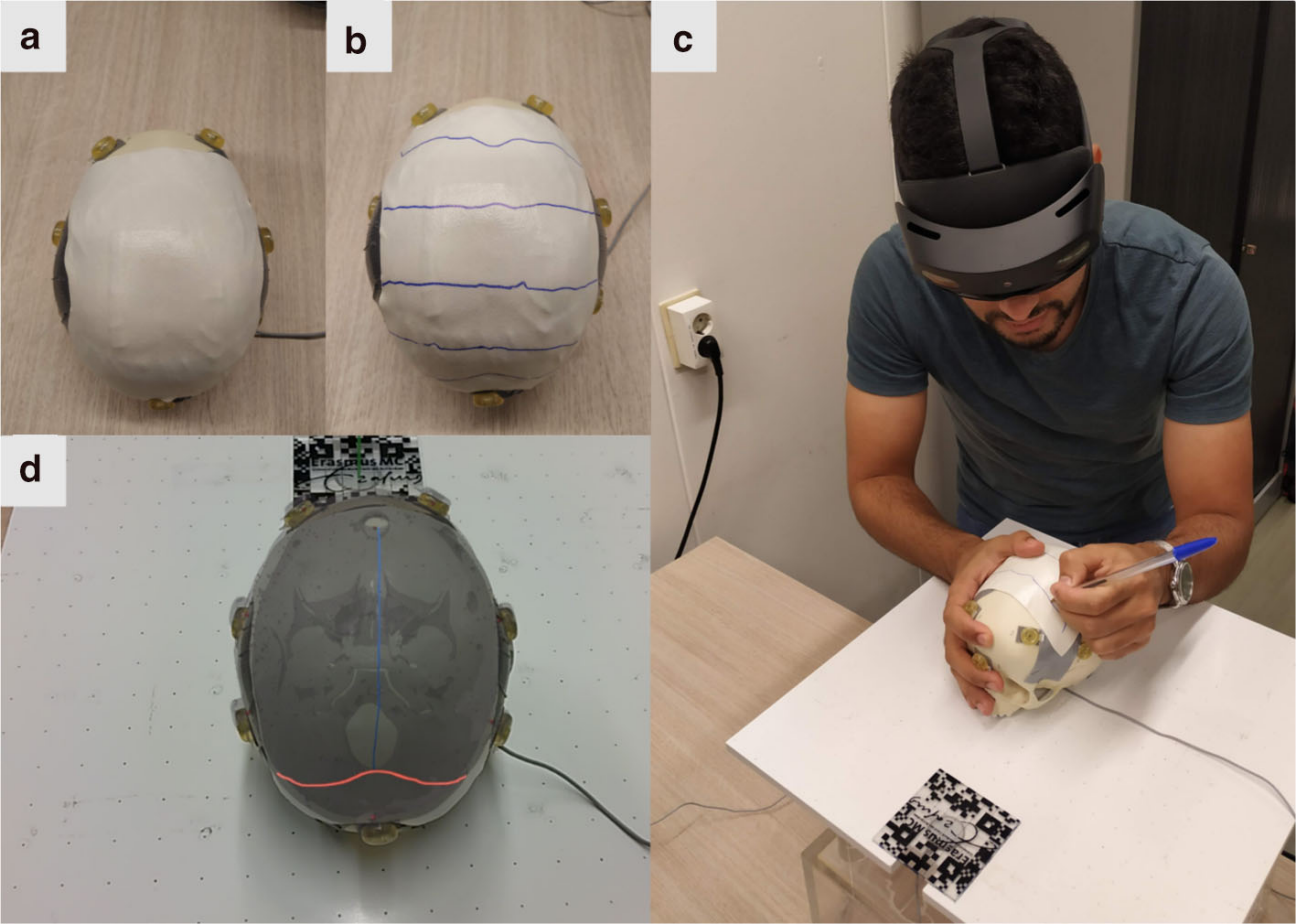
Sutures delineation on phantoms: **a** SK2 covered with tape and surgical foam, **b** delineated sutures by the participant, **c** the delineation task, **d** view of the HL2 during the experiment with lambdoid (red) and sagittal (blue) sutures shown

Before conducting the experiment, the skull surface was taped and covered with a 1-mm-thick surgical foam to prevent any visual indications of the sutures’ locations. Figure 5a shows the skulls after preparation for the experiment. The EM-patient sensor was also rigidly attached on the right side of each skull (behind the eye) and was kept in place during the whole study.

A number of participants volunteered for this study. Each participant was first asked to sign a consent form for participation after which the experimental protocol was explained. Next, a model alignment was performed by the operator (not the participants) using the anatomical landmarks defined in the preplanning step (see “Preoperative planning” section).

For each participant, eye-calibration (a standard procedure for users using the HL2 for the first time) was conducted to adjust the HL2 for their eyes. The experiment then started with a brief training session where the participants were shown the skull model aligned on the physical phantom, with three virtual sutures projected on the skull surface. In this way, they were introduced to using the HL2.

After the training session, the participants start the delineation task, where for each visualized suture they were asked to delineate it on top of the surgical foam using a pen of 0.8 mm tip size. The sutures were visualized one by one and controlled by an operator that guided the participants through the experiment. Figure 5 shows the delineation task. Note that the sagittal suture was only visualized to the participants for center-line guidance and not included in the delineation task. That is because it is usually easy to locate in craniosynostosis surgeries and no accurate locating is required. A sample video of the sutures visualized in the HL2 can be seen in Online Resource 1 (see supplementary material).

During the experiment, the time required for suture delineation was recorded starting from the time the suture is shown to the participant until the suture is delineated (visualization time) as well as the time took for the actual delineation (delineation time).

After the delineation of the five sutures on each skull, the experiment ended and the participants were asked to fill-in a system usability scale (SUS) questionnaire [10] in addition to some questions about the visualization and model alignment.

**Fig. 6:**
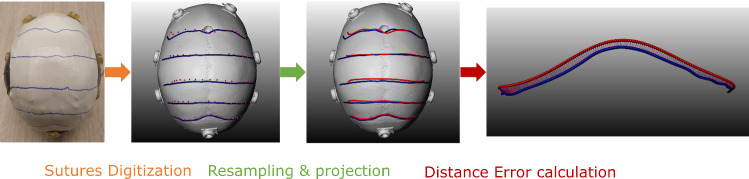
Followed steps for digitization of the drawn sutures and the calculation of distance error between drawn (red) and CT-annotated (blue) sutures

### Evaluation

#### Registration

Since the model-to-patient registration plays a crucial role and is one of the main factors contributing to the overall suture delineation error, we first assess the registration performed with the anatomical landmarks that was used for the experiment. To this end, we calculate the fiducial registration error (FRE), which is the RMSE of the point-based rigid registration between the anatomical landmarks annotated in CT and the same landmarks digitized using the EM tracking system. In addition, we compute a target registration error (TRE) using the conical markers attached to the skull.

#### Suture delineation

After the delineation of the sutures by each participant, an independent optical tracking system (OTS) (NDI Vega, Waterloo, ON Canada), that is not part of our clinical system setup, was used for the evaluation of the delineated sutures, to avoid any bias that may be in the tracking of the EMTS. For that, a rigid-body marker was attached to each skull phantom and a second model alignment was performed using the conical markers attached to the skull. A tracked pointer was then used to digitize the delineated sutures and bring them to the CT space using the obtained registration matrix.

To compare the delineated sutures to the planned sutures (annotated in CT), the digitized suture lines are projected on the skull surface and resampled. Subsequently, the surface area $S_A$ spanned between the overlapping parts of the CT-annotated and digitized sutures is computed (see Fig. 6). The distance *d* between the CT-annotated sutures and the drawn sutures is then calculated as follows:

1 $$\begin{aligned} d = S_A / D_L, \end{aligned}$$

where $D_L$ is the length of the overlapping part of the two sutures.

**Table 1:** Participants demographics

All participants ($n = 12$)	
*Age*	
20-30	42%
30-40	33%
50+	25%
*Background*	
Medical	25%
Technical	75%
*Familiarity with the HoloLens*	
Yes	25%
No	75%

**Table 2:** Mean and standard deviation of the registration error (mm) for the experiment and evaluation registration for both SK1 and SK2

	Experiment registration error		Evaluation registration error
Skull phantom	EMTS FRE	EMTS TRE	OTS FRE
SK1	$0.52\pm 0.08$	$1.88\pm 0.41$	$0.61\pm 0.05$
SK2	$0.45\pm 0.07$	$1.84\pm 0.43$	$0.70\pm 0.05$

**Table 3:** Mean and standard deviation of distance error (mm) for the delineated sutures: coronal, virtual and the lambdoid sutures for both SK1 and SK2

Skull phantom	Coronal	Virtual	Lambdoid	All
SK1	$1.96\pm 1.03$	$2.30\pm 0.86$	$3.15\pm 1.57$	$2.40\pm 0.89$
SK2	$1.97\pm 0.63$	$2.23\pm 1.04$	$3.10\pm 1.43$	$2.35\pm 0.88$

## Results

In this study, twelve participants were involved. Table 1 shows the demographics of the participants, with 75% ($n=9$) of the participants using the HL2 for the first time. 25% ($n=3$) of the participants have a medical background (two of which are surgeons), while the rest have a technical background.

### Registration

Table 2 shows the registration error (FRE and TRE) using the anatomical landmarks (experiment registration) as well as the registration error for the OTS using the conical landmarks (evaluation registration).

### Suture delineation

**Fig. 7:**
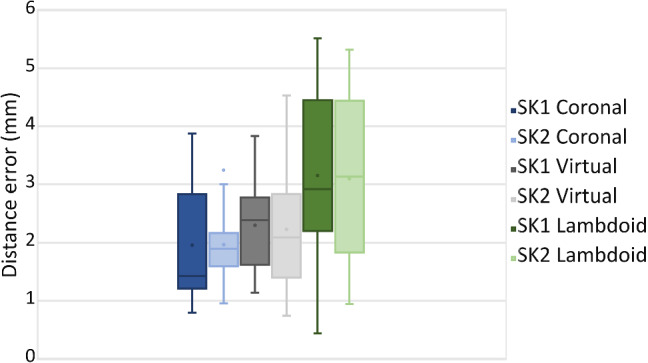
Boxplot of the distance error (mm) for the delineated sutures for SK1 and SK2

The distance error of the delineated sutures with respect to the target planning (CT-annotated) is shown in Table 3. For both skulls, an average delineation distance error of 2.4 mm is observed, with a mean error of around 2 mm for the coronal, 2.3 mm for the virtual sutures and 3.1 mm for the lambdoid. A boxplot of the results is also shown in Fig. 7.

### Delineation time

Table 4 shows the average time took the participants to visualize the sutures for delineation and to delineate them on the skull phantoms.

### Usability questionnaire

The AR system for craniosynostosis achieved a $73\pm 12$ SUS usability score, where most participants found the system easy and simple to use. In addition to the SUS, additional questions about the visualization and model alignment were asked. Table 5 shows the questions and the responses of the participants. Most participants found the skulls and sutures visualization satisfactory, and they perceived the virtual models well aligned. However, some participants reported a movement of the virtual model visualization during the experiment.

**Table 4:** Time took by the participants for suture visualization and delineation

Skull phantom	Visualization time (s)	Delineation time (s)
SK1	$23\pm 9$	$13\pm 5$
SK2	$21\pm 8$	$14\pm 5$

**Table 5:** Visualization and alignment questions asked to participants

Question	SD	D	N	A	SA
I think the visualization was clear and intuitive				25%	75%
I perceived that the virtual models were well aligned with the physical phantom		8.5%	8.5%	33%	50%
The virtual models were generally stable			25%	59%	16%

*SD* strongly disagree, *D* disagree, *N* neutral, *A* agree, *SA* strongly agree

## Discussion

The AR visualization of cranial sutures led to a mean delineation error of around 2.4 mm for both skulls, which shows the generalizability of the system over anatomical changes. The achieved accuracy indicates the usability of the developed AR-EM system to assist in the planning of spring-assisted minimally invasive craniectomy. It shows that the system can help in locating the cranial sutures with an acceptable error (less than 5 mm). We can see from the results in Table 3 that delineation of the coronal suture achieved the lowest distance error (around 2 mm on average), followed by the virtual sutures, then the lambdoid suture with around 3.3 mm. The increase in distance error can be attributed to the registration error of the anatomical landmarks which are located at the front side of the skulls near the coronal suture and further from the lambdoid suture. As shown in Table 2, the TRE of the EMTS registration was as high as 2 mm which would contribute to the overall distance error between delineated and originally planned sutures.

In this phantom study, the anatomical landmarks were chosen similar to the navigation procedure in craniosynostosis where landmarks are annotated around the face, which is the region with the most prominent anatomical features. Note that in the OR, with the presence of skin, attention should be paid to ensure good patient alignment. Since some of the participants in our study were not surgeons and have no prior experience with point-based registration, we opted for the operator to perform patient alignment. However, we expect the results to be relevant since registration-based on anatomical landmarks is a common procedure in neuro-navigation. In the OR, landmarks that are close to the target lambdoid suture such as the mastoid and inion can be included to improve patient alignment.

The results shown in Table 3 and Fig. 7 show a high standard deviation in the distance error of the delineated sutures. This can possibly be attributed to when sometimes the QR-code is occluded or out of sight during the experiment, causing the virtual model to be displaced. A couple of users during the experiment reported a shift and some movement of the virtual model when the QR-code is occluded. This can be addressed by using multiple QR codes or a cubic QR code that can be viewed from different directions. The users also reported a difficulty in viewing the tip of the pen during delineation due to the AR overlay. A possible solution would be to track the pen and show its overlay [11], or to dim and control the sutures overlay around its tip. However, as indicated in Table 5, most participants reported perceiving the visualization of the skull and sutures satisfactory.

The developed AR-EM system although does not improve on the setup time, it offers a more intuitive and in-situ 3D visualization compared to traditional navigation systems. Moreover, combining the HoloLens with an EMTS alleviates the need to maintain a direct line-of-sight with the patient tracking sensor, which is required in inside-out marker-based tracking. With such combined AR-EM navigation system, patient tracking can be more reliable with no additional perceived latency for communication.

The conducted study, although it was evaluated on physical phantoms, it was designed to reflect the clinical setup in the OR, taking into account the patient position, attachment of the patient sensor, system setup, surgery procedure, and accessibility of anatomical landmarks for registration. We believe the obtained delineation accuracy in this study can be achieved in the OR with a few considerations in mind. These include good patient alignment for accurate visualization, and a stable marker tracking for reliable navigation. Clinical validation of the system with real patients is to be addressed in a future work.

## Conclusion

This study demonstrates that an AR-EM system has sufficient accuracy to be used for surgical guidance in minimally invasive spring-assisted craniectomy. For 120 delineated sutures, an average distance of of 2.4 mm with respect to the planned sutures was obtained. The system can help surgeons accurately locate cranial sutures in the OR. Further improvements on the QR-code tracking stability and registration accuracy would help in bringing this approach to clinical applications.

## Supplementary Information

Below is the link to the electronic supplementary material.

Supplementary file 1 (mp4 45067 KB)
